# 3did: a catalog of domain-based interactions of known three-dimensional structure

**DOI:** 10.1093/nar/gkt887

**Published:** 2013-09-28

**Authors:** Roberto Mosca, Arnaud Céol, Amelie Stein, Roger Olivella, Patrick Aloy

**Affiliations:** ^1^Joint IRB-BSC Program in Computational Biology, Institute for Research in Biomedicine (IRB Barcelona), c/ Baldiri Reixac 10-12, 08028 Barcelona, Spain, ^2^Center for Genomic Science of IIT@SEMM, Istituto Italiano di Tecnologia (IIT), Via Adamello 16, 20139 Milan, Italy, ^3^California Institute for Quantitative Biomedical Research (qb3) and Department of Bioengineering and Therapeutic Sciences, MC 2530, University of California San Francisco (UCSF) CA 94158–2330, USA and ^4^Institució Catalana de Recerca i Estudis Avançats (ICREA), Passeig Lluís Companys 23, 08010 Barcelona, Spain

## Abstract

The database of 3D interacting domains (3did, available online for browsing and bulk download at http://3did.irbbarcelona.org) is a catalog of protein–protein interactions for which a high-resolution 3D structure is known. 3did collects and classifies all structural templates of domain–domain interactions in the Protein Data Bank, providing molecular details for such interactions. The current version also includes a pipeline for the discovery and annotation of novel domain–motif interactions. For every interaction, 3did identifies and groups different binding modes by clustering similar interfaces into ‘interaction topologies’. By maintaining a constantly updated collection of domain-based structural interaction templates, 3did is a reference source of information for the structural characterization of protein interaction networks. 3did is updated every 6 months.

## INTRODUCTION

Proteins are key players in virtually all events that take place within and between cells. However, they seldom act alone and it is their complex interrelationships that will ultimately determine the behavior of a biological system. For this reason, large efforts have been devoted to unveiling the complex network of interactions between proteins underlying biological processes, producing large interactomes for several organisms, including human (1–3). High-throughput interaction discovery experiments provide valuable information as to who-interacts-with-whom but, to fully understand how protein interactions occur, we need to incorporate high-resolution molecular/atomic details, which are currently available in the Protein Data Bank [PDB, (4)].

Several efforts over the last years aimed at mining the data in the PDB to provide a comprehensive structural characterization of protein interaction networks (5–7). While these studies took different approaches they all agree on one point: interactions are often achieved by the reuse of evolutionary conserved structural modules, represented by domain families. Domains can be found in interaction with other domains (domain–domain interactions or DDIs) or with short, usually structurally extended peptides described by a recurring motif of amino acids (domain–motif interactions or DMIs). The possibility of producing a complete and exhaustive mapping of structural data on protein interactomes depends, therefore, on the availability of a reliable and extended catalog of domain-based 3D structural templates. Given the rate at which new interactions are discovered and new structures of complexes are experimentally characterized, it is paramount for this catalog to be constantly updated.

Several bioinformatics studies have attempted to define and classify domain interactions, both DDIs (8–13) and DMIs (14–17), and produced databases of domain-related interaction models but many of them are not regularly updated or are not available anymore. All these databases also vary in the way they define domains. Some of them use the definition provided by SCOP (18) or CATH (19), which are based on the analysis of experimental structures and are known to lag behind the status of PDB by several years.

The database of 3D interacting domains (3did) is a collection of 3D structures of domain-based interactions, both DDIs and DMIs, based on domain definitions from Pfam (20), ensuring a higher coverage of the protein sequences universe. It has been constantly available to the scientific community for more than 8 years (21–23). With the current version it integrates a pipeline for the automatic identification of novel domain–peptide interactions. Periodic updates will be performed every 6 months to reflect the latest contents of the PDB and the latest definitions of domain families from Pfam. All these characteristics make 3did a reference catalog of domain-based interaction and an essential component for the structural characterization of protein interaction networks.

## DOMAIN–DOMAIN INTERACTIONS

DDIs occur when two globular domains form a stable interface. Interfaces in DDIs are usually relatively large [2000 Å^2^ on average (24)]. Several possible definitions of domains are available based on conserved globular structure or on evolutionary conserved residue sequences. For instance, SCOP (18) and CATH (19) are two catalogs of structurally conserved domains. In 3did we use the domains definitions provided by Pfam, generated from representative homologous protein that are searched against large datasets of protein sequences. Pfam domains, being defined on evolutionary conserved modules at the sequence level, have the advantage of showing a higher coverage of the sequence space. Due to the faster collection of protein sequences, Pfam definitions are updated more often than structure-based domain definitions. The current version of 3did uses Pfam version 27.0, which includes more than 14 000 domain families. Domains are searched on the sequences of all chains present in the PDB by using the pfam_scan.pl script provided by Pfam [which uses HMMER3 (25)]. All nonoverlapping hits are retained. In case of pairs of domains where one overlaps with the center (in sequence) of the other, only the domain with the highest score is retained. We exclude chains shorter than 11 residues, chains reporting only the position of Cα atoms and those where only the backbone has been traced.

We estimate the number of residue–residue interactions between pairs of contacting domains either within the same chain (intrachain) or between two different chains (interchain). We require at least five estimated contacts [hydrogen bonds, electrostatic or van der Waals interactions, as described in (26)] in order to account for an interaction between the two domains. Finally we assign a *z*-score to the DDIs [based on (26,27)]. For every pair of interacting domains, we cluster the corresponding structural templates on the basis of the interaction interface in order to characterize different modes of interaction between the same pair of domains, as described previously (23).

**Figure 1.**
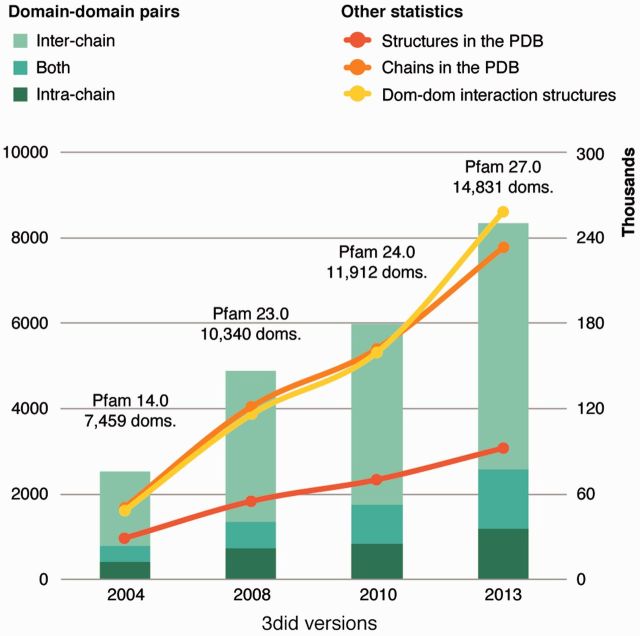
Growth of 3did throughout its four releases. The colored bars represent the number of DDI pairs with only intrachain structural templates (dark green), only interchain templates (medium green) and both types of templates (light green). Bar plots refer to the *y*-axis on the left. The lines represent the growth in the number of structures (dark orange) and chains (light orange) in the PDB. The yellow line represents the number of domain–domain structural templates in 3did (i.e. the number of redundant structural instances of DDI). Line plots refer to the *y*-axis on the right.

The current version of 3did contains 258 079 structural instances of DDIs of which 68 861 are intrachain and 189 218 are interchain. These correspond to 8328 unique domain–domain pairs (1190 with only intrachain instances and 5747 with only interchain instances while 1391 have both intra and interchain instances). With respect to the last version of 3did (2011) we observed an impressive growth of 62% in the number of DDI structures corresponding to 39.5% more domain–domain distinct (i.e. nonredundant) pairs, reflecting the constantly increasing rate of growing of the PDB and Pfam (Figure 1; please note that we have introduced a release numbering scheme based on the year and month of release: the current version is 2013_06). Table 1 reports the top 10 domains ranked on the number of partner domains. The table also shows that the PDB contains highly redundant data for DDIs. In fact, for every pair of interacting domains, usually there are several structural instances of that DDI, showing, in many cases, different interaction topologies and, sometimes, multiple instances for the same topology.

**Table 1.** Top 10 interacting domains with the corresponding number of protein partners. DDI pairs in 3did have variable numbers of structural templates. For example, even if the C1-set domain has less interacting domains than the V-set domain, it has many more redundant structural templates in the PDB

Domain name	Pfam id	# partners	#interaction structures
V-set	PF07686	161	8962
Ras	PF00071	62	610
Pkinase	PF00069	54	1888
Trypsin	PF00089	50	1753
ubiquitin	PF00240	43	632
C1-set	PF07654	39	9114
WD40	PF00400	32	2205
EF-hand_7	PF13499	32	713
Ig_2	PF13895	29	312
Ank_2	PF12796	29	428

## DOMAIN–MOTIF INTERACTIONS

**Figure 2.**
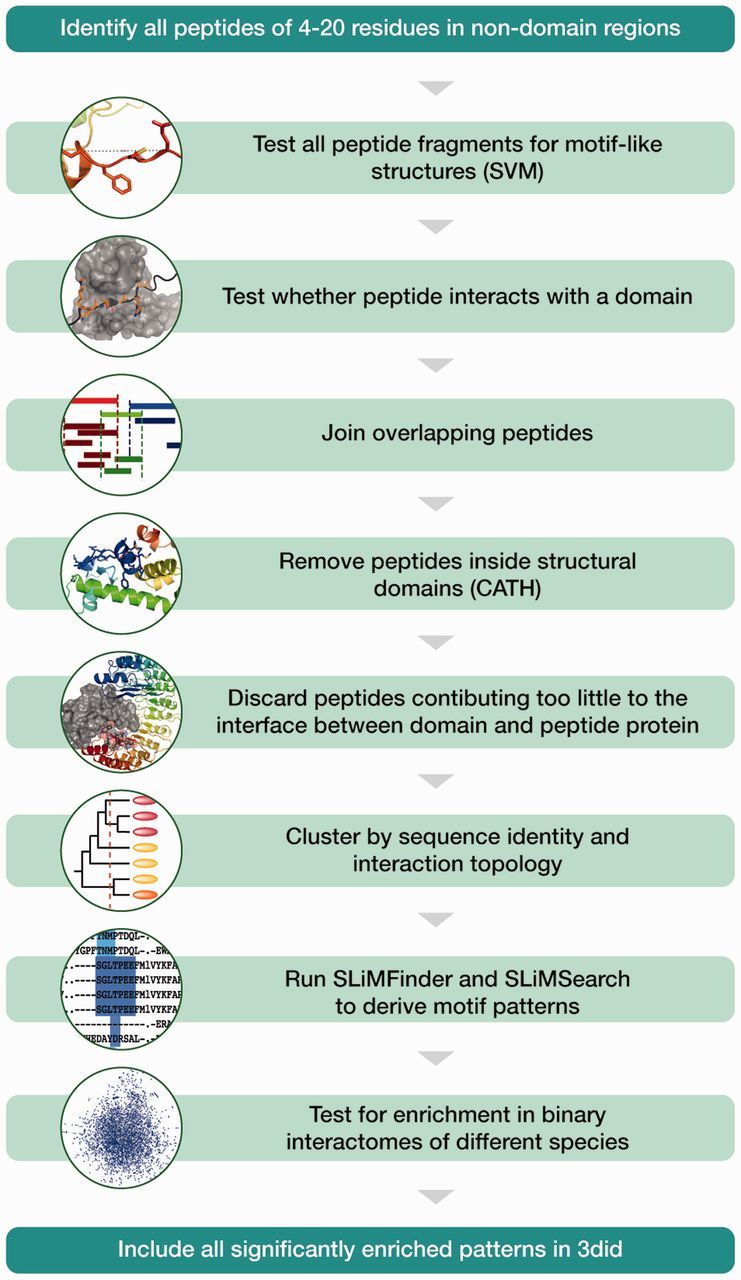
Overview of the DMI discovery pipeline. The main steps of the DMI discovery pipeline are outlined, with filtering steps to remove spurious hits. Details can be found in (33).

Domains have also been observed to bind short linear motifs, which show considerably smaller interfaces than those in DDIs [350 Å^2^ on average (24)]. Given the smaller interface, DMIs are often weaker in nature and thus often used in transient associations such as signaling networks (28). Only a small number of key residues are required for binding, allowing fast evolution of these interactions (29). However, the short motifs are harder to detect automatically than evolutionary conserved domain fingerprints, therefore many resources of domain–motif interactions, such as ELM (30), rely on manual curation. Interactome-wide approaches rely on motifs and protein interaction data to suggest DMIs [e.g. (31)]. As an alternative approach to DMI detection, commonly observed structural features of these interactions have enabled automated searches in the PDB (32,33). In both interactome- and structure-based approaches, the main challenge is to separate spurious hits from truly over-represented domain–motif pairs. This is usually performed by calculation of statistical significance against a random background as well as enrichment in alternative datasets, such as interactomes of different species. The approach now included in 3did has been described in detail (33) and is outlined in Figure 2. Previous versions of 3did reported only one motif for each DMI topology, even if multiple were found to be significant, while we now report all significant motifs.

The DMI-collection in 3did now contains peptides binding to 113 distinct domains, an increase of ∼2.5-fold over the 46 domains described in our 2010 article (33). This goes along with a ∼3-fold increase in structures of DMIs, from 1500 to 4500. Since the discovery of novel DMIs requires intensive computation, we have decided to rebuild the contents of the database every 6 months, synchronized with the update of our other structural database Interactome3D [http://interactome3d.irbbarcelona.org, (6)].

## 3did NEW INTERFACE

The web interface has been entirely redesigned to allow an easier and more enjoyable search. The home page displays basic statistics about the domains and motifs present in the database and informs the user about the versions of Pfam and PDB that are currently used. The results in the database may vary from one version to the other and the user should be aware of each update. The home page also permits a simple query to 3did for a domain or a motif. The different search tools available in the previous version of 3did have been grouped in a single search page. This page allows to search for a domain (either the name of the domain or its Pfam accession number can be used), a motif name, a structure (by PDB ID) or any term from the Gene Ontology (34). The association between Pfam accessions and GO terms is downloaded from the Gene Ontology website (http://www.geneontology.org). Alternatively it is possible, through the ‘browse’ tab, to browse all domains and motifs present in 3did or to explore a GO tree and retrieve all the domains associated to any GO term (Figure 4C).

**Figure 3.**
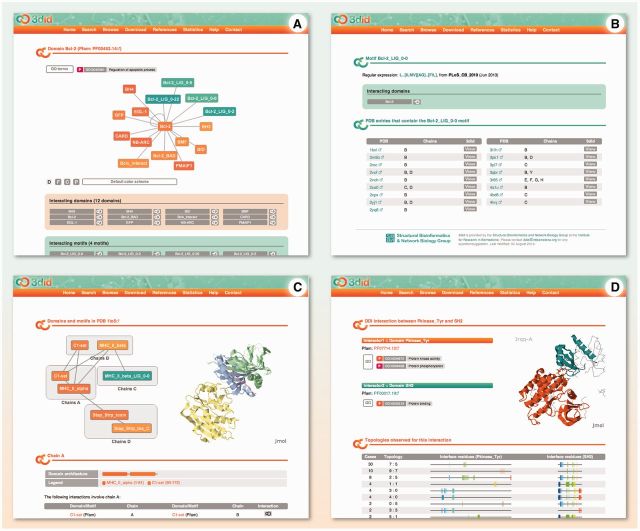
Views available in 3did. 3did provides four views to browse the data contained in the database: the Domain view (**A**), the Motif view (**B**), the PDB view (**C**) and the Interaction view (**D**).

**Figure 4.**
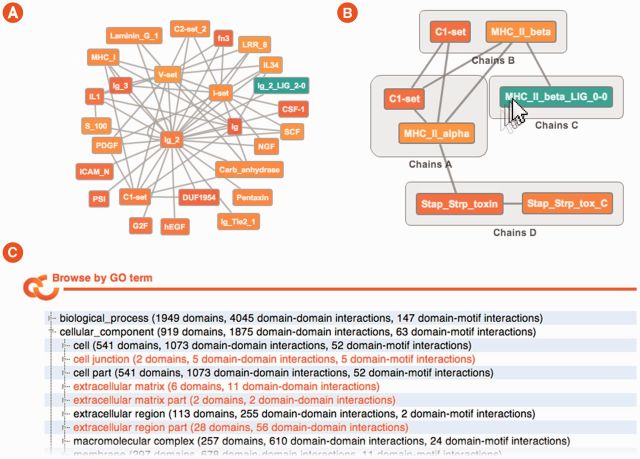
Browsing 3did. (**A**) Interactive view of the DDI and DMI network involving a particular domain. In orange are the domains while the motifs are in green. By clicking on any node or edge you are redirected to the page showing details about the corresponding domain, motif or interaction. (**B**) Interactive view of the domains and motifs in a PDB file. It shows the domain composition of the different chains (clustered on identical domain composition) as well as the motifs present in the chains. Lines connect domains and motifs that are interacting. Both nodes and lines can be clicked in order to visualize the details of the corresponding domain, motif or interaction. The CytoscapeWeb-based network visualizations (A and B) require a Flash plug-in to be installed in the browser to run. (**C**) Browse by GO term. A new tree view in the ‘Browse’ page allows searching for all the domains that are annotated with a specific GO-term.

The data in 3did are displayed in four different views: the domain, motif, interaction and PDB views (Figure 3). The domain view is composed of three parts. The first part shows, both graphically and as a list, the domains and motifs that interact with the query domain. In the graphical interface, based on CytoscapeWeb (35), the interacting domains are displayed in orange and the interacting motifs in green (Figure 4A). A set of four buttons below the graph allows updating the network and displaying the GO terms associated to each domain. The interacting domains and motifs are also displayed as a list. Both lists and the network are linked to the page describing the domain or motif and the corresponding DDIs or DMIs. The second part of the page displays the residues that are involved in the interactions and the third one lists the structures in which the interactions have been identified and the chains that are involved. The second view, the motif view, lists the interacting domains and the structures in which the interactions have been identified. The third view, the interaction view, displays in a Jmol applet (http://www.jmol.org/) the first structure (in alphabetical order) in which the interaction has been identified. Different interfaces, involving different residues, may be identified for each interaction. Those different interfaces, or topologies, are also listed in the interaction page, with the residues involved. In addition, the list of structures in which the interaction has been found is provided: each structure can be displayed in Jmol by clicking the associated ‘View’ button. Finally, the PDB view allows displaying all domains and motifs identified in a specific PDB structure as well as their interactions. The DDIs and DMIs are represented as a network in CytoscapeWeb and the structure is displayed in Jmol. In addition, the domain architecture of each chain in the PDB file is listed, as well as the interactions in which each domain of the respective chain is involved (Figure 4B).

The navigation from one view to the others is facilitated by a number of links, including the clickable domains and motifs names, the DDI and domain–motif interaction buttons, the ‘View’ and ‘Jmol’ buttons, and the nodes and edges in the networks. The help page contains an illustrated description and additional information on how to browse the 3did web site.

## CONCLUDING REMARKS

Full atomic characterization of protein–protein interaction at the ’omics level is becoming an impending need in the everyday work of biologists (36). Many different approaches have been taken in order to achieve this. Most of them exploit the observation that evolutionary conserved domain families are used as independently interacting modules in proteins. These functional modules are reflected at the protein structural level and are involved in a complex network of interactions for which high-resolution structures are available in the PDB. 3did collects and organizes the catalog of these structures both for DDIs and DMIs. Furthermore, it makes the catalog available to the scientific community through an intuitive web interface for browsing the data and through batch downloads that enable the use of the data in large-scale bioinformatics studies. By providing a constantly updated, extensive catalog of 3D structures of domain-based interactions, 3did aims to be a reference resource for the structural annotation of protein interaction networks.

## AVAILABILITY

3did can be accessed interactively from the web pages at http://3did.irbbarcelona.org, where it is also possible to download the full dataset in tab delimited files or in a full mysql dump that can be restored locally.

Four tab delimited files are available: 3did_flat.gz contains interacting domain pairs and the instances of these interactions in PDB structures, 3did_dmi_flat.gz contains DMIs, i.e. motifs with the corresponding pattern as well as all 3D instances of the interaction, 3did_interface_flat.gz contains the different binding topologies and 3did_global_interface_flat.gz contains the global interfaces.

More information about the download formats is available in the download page. 3did, including both DDIs and DMIs, will be updated twice per year with the latest versions of PDB and Pfam.

## FUNDING

Funding for open access charge: The Spanish Ministerio de Ciencia e Innovación through the grant [BIO2010-22073] and the European Commission under FP7 Grant Agreement [306240 (SyStemAge)].

*Conflict of interest statement*. None declared.
